# Brain Functional Connectivity Changes After Transcranial Direct Current Stimulation in Epileptic Patients

**DOI:** 10.3389/fncir.2018.00044

**Published:** 2018-05-30

**Authors:** Franca Tecchio, Carlo Cottone, Camillo Porcaro, Andrea Cancelli, Vincenzo Di Lazzaro, Giovanni Assenza

**Affiliations:** ^1^Laboratory of Electrophysiology for Translational neuroScience, Institute of Cognitive Sciences and Technologies, National Research Council, Rome, Italy; ^2^Movement Control and Neuroplasticity Research Group, Department of Kinesiology, KU Leuven, Leuven, Belgium; ^3^Department of Information Engineering, Università Politecnica delle Marche, Ancona, Italy; ^4^Unit of Neurology, Neurophysiology, Neurobiology, Department of Medicine, Università Campus Bio-Medico di Roma, Rome, Italy

**Keywords:** epilepsy, drug-resistant, transcranial direct current stimulation (tDCS), functional connectivity, electroencephalography

## Abstract

Focal epilepsy is a network pathology, where the brain connectivity of the epileptic focus (EF) influences seizure frequency and cortical dysfunction. Growing evidence supports a clinical efficacy of cathodal transcranial direct current stimulation (ctDCS) in drug-resistant epilepsy (DRE). ctDCS effects can be merely attributed to the inhibition of cortical excitability, which is abnormally increased in epilepsy, but its effect on brain network of DRE patients has never been reported. We aimed at exploring the hypothesis that functional connectivity (FC) changes may explain part of ctDCS clinical effects in DRE patients. We assessed the ctDCS-induced changes of electroencephalography-derived brain FC of a group of six temporal lobe DRE patients receiving a seizure reduction after ctDCS. By a single-subject eLORETA analysis, we compared the FC among the EF region and other nine bilateral macro-regions, before and after Real and Sham ctDCS in a double-blind Sham-controlled crossover design. FC changed after Real ctDCS in all patients despite no appreciable changes occurred after Sham. Most of FC changes (73%) involved the EF region. The epileptic seizure reduction correlated with the increase of the EF FC, in the whole frequency band and in the theta band. This small-sample analysis clearly revealed that ctDCS induced FC changes in the brain network of temporal lobe DRE patients. Our data support the hypothesis that FC changes may contribute to explain the effects of ctDCS in epilepsy, offering a new scenario in the personalization of neuromodulation interventions in epileptic people.

## Introduction

The brain is a network in nature, thus the understanding of neurological disorders requires to go beyond the selective study of the structural alterations and to deal with its short- and long-range connections. The modern use of electroencephalography (EEG) and functional magnetic resonance imaging allows an accurate quantification of neural structural and functional connectivity (FC) and has confirmed the old clinical evidence of the occurrence of neurological deficits involving areas far from the lesion (Nickels et al., 2016). FC alterations play a crucial role in focal epilepsy. Actually, recent neuroimaging studies confirmed that focal epileptic neurons create an own pathologic network and its activity can interfere with other cortico-subcortical networks causing a broad spectrum of cognitive disabilities (Rektor et al., 2013; Moshé et al., 2015; Englot et al., 2016; Výtvarová et al., 2017). Furthermore, epileptic activity drives a certain degree of plastic reorganization of brain networks (Englot et al., 2015; Gleichgerrcht et al., 2015). This re-organization can be clinically relevant, as it can improve the accuracy of the diagnosis (Douw et al., 2010), helps the pre-surgical planning (Van Dellen et al., 2014), and represents a marker of the efficacy of surgical therapies (Douw et al., 2008). Thus, the research of innovative clinical interventions able to interact specifically with the epileptic network may positively affect the quality of life of epileptic patients. Actually, one-third of epilepsy patients develop drug resistance, and only half of them could benefit from the surgical removal of epileptic focus (EF). Neuromodulation represents the only therapeutic chance for the remaining patients (Lee et al., 2012; Capone et al., 2015; Assenza et al., 2017b), but the approved devices require a surgical implantation of the stimulators. Transcranial direct current stimulation (tDCS) is a non-invasive neuromodulation technique, which in the last two decades was used efficaciously against neurological and psychiatric sufferance. Positive effects were induced to support motor function in stroke patients (Boggio et al., 2007; Hesse et al., 2011), against depression (Fregni et al., 2006; Nitsche et al., 2009) and pain (Zhu et al., 2017), and against alcohol or smoke craving (Boggio et al., 2008; Fregni et al., 2008). Intervention durations, with 15–20 min per day, ranged from 2 to 30 days, most commonly five consecutive days in clinical context. Sample size in the clinical trial range from about 10 persons to 40 depressed (Boggio et al., 2008) and 64 persons studied with respect to decision making behavior (Knoch et al., 2008). Cathodal tDCS is able to inhibit cortical excitability, which is abnormally increased in epilepsy (Liebetanz et al., 2002), and to interfere with FC (Polanía et al., 2011; Antonenko et al., 2017; D’Mello et al., 2017; Krause et al., 2017). A recent study suggests a clinical efficacy of ctDCS applied over the EF in reducing seizure frequency in focal temporal lobe drug-resistant epilepsy (tDRE) (Assenza et al., 2017a).

Here, we aimed at investigating whether ctDCS-related FC changes occur together with ctDCS clinical efficacy in DRE patients.

## Materials and Methods

Brain FC changes induced by ctDCS were studied in six patients affected by tDRE (clinical data in **Table 1**), who are part of the sample treated by Assenza et al. (2017a) in a randomized double-blind sham-controlled crossover trial demonstrating the clinical efficacy of ctDCS in tDRE. Inclusion criteria: age > 18 years; temporal DRE; mean seizure frequency (SF) ≥ 2 week in the last 3 months; patients or caregivers are able to reliably provide seizure diary. Exclusion criteria: psychogenic seizures; multifocality; major psychiatric, or neurological disorders other than epilepsy; electrical medical devices. All patients signed a written informed consent to participate to this study approved by the Ethics Committee of the Campus Bio-Medico University (UCBM).

**Table 1:** Demographical and clinical data of participants.

ID	Gender	Age (years)	Diagnosis	Seizures type	EF	Years from 1st seizure	AEDs (mg/die)
S.C.	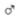	24	Cryptogenic	CP	T4	20	FLB 2400 LTG 400
A.C.	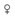	50	Cryptogenic	CP+SG	F8	45	CBZ 1400 VPA1800
C.T.	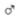	17	Symptomatic (diabetes type I, brainstem atrophy, retinitis pigmentosa)	CP+SG	F7	10	PB 100 CBZ 1200 CLB 20
S.B.	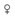	23	Symptomatic (meningitis)	CP+SG	T4	22	PB 100 LEV 3500
L.P.	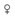	53	Symptomatic (delivery problems)	CP+SG	T4	49	LTG 150
I.C.	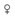	36	Cryptogenic	CP	T5	31	CBZ 400 TPM 100


*EF, EEG epileptic focus, site of ctDCS; CP, complex partial; SG, secondarily generalized; AED, anti-epileptic drugs; FLB, felbamate; LTG, lamotrigine; CBZ, carbamazepine; VPA, valproic acid; PB, phenobarbital; LEV, levetiracetam.*

**FIGURE 1:**
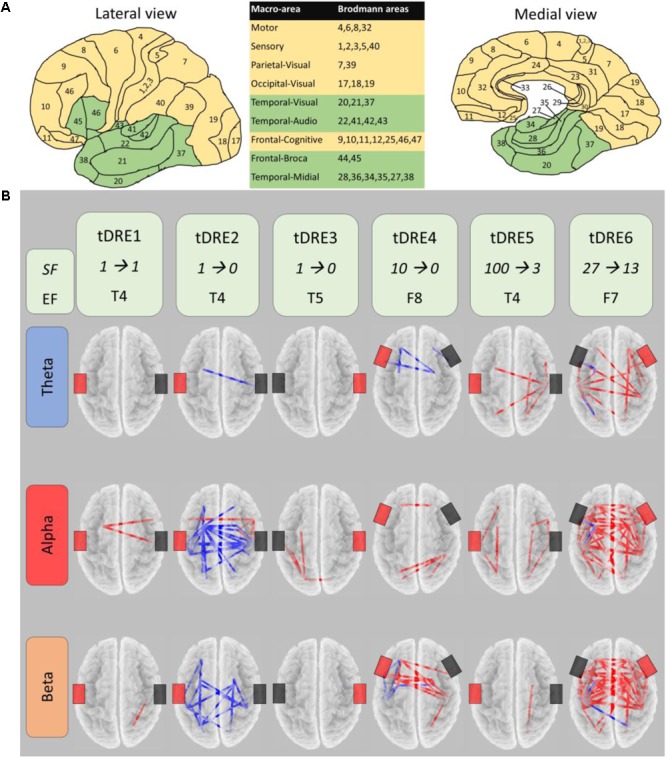
CtDSC-induced changes of the brain functional connectivity. **(A)** Lateral and medial views of the nine regions per hemisphere selected for functional connectivity (FC) analysis (macro-areas, included Brodmann areas listed in table). Epileptic focus macro-areas in green. **(B)** For each temporal lobe drug-resistant epileptic patient (tDRE), representation of the FC connectors changed in post- with respect to pre-ctDCS in the three frequency bands. Red (blue) connectors indicate increased (decreased) FC. For each tDRE, we indicated the seizure frequency (SF) in the week pre- and post- ctDCS as well as the epileptic focus location according to the EEG 10–20 International System. Black and red rectangles indicate respectively the cathode and the anode of the ctDCS.

We chose those patients having ≥1 h of EEG recording free of artifacts impairing subsequent EEG analysis for each condition (pre- and post-sham, pre- and post-ctDCS). Each patient underwent to both ctDCS and Sham, 1-month apart, according to a randomized crossover design. One mA ctDCS was delivered for 20 min with a battery-driven stimulator (Schneider Electronic, Gleichen, Germany-Newronika) connected to a saline-soaked pair of surface sponge conductive electrodes (5 cm × 7 cm). Cathodal electrode (reducing cortical excitability) was placed over the EF, defined by clinical and scalp interictal EEG data, and anodal electrode on the opposite homologous region. To study the ctDCS-modulated EEG-derived (19-channels, Micromed, Italy) brain FC, we collected the resting state signal for 1 h just before and after Real and Sham stimulations. After artifact removal without epoch exclusion via independent component analysis (Barbati et al., 2004), we estimated the FC in theta (4–8 Hz), alpha (8–13 Hz), and beta (13–30 Hz) frequency bands using the exact low-resolution electromagnetic tomography (eLORETA) software^1^. We identified 10 homologous functional macro-areas in the brain (**Figure 1A**), one of which included all the EF (green in **Figure 1**) of our patients. We estimated the FC between the eLORETA current density time series of each macro-area by the Lagged Linear Coherence (LagR) algorithm, implemented in eLORETA, as a measure not affected by volume conduction and by the low spatial resolution (Pascual-Marqui, 2007). We windowed the 1-h recording, selecting a fix number of 3 min-epochs, and submitted the FC estimated in each of the 18 epochs to a single-subject analysis comparing the pre- vs. post-ctDCS FC. The 1-h recordings before and after the tDCS treatment allow an analysis with the proper statistical power to assess the tDCS-induced changes in resting state FC by eLORETA estimate in each single-subject. The previous study on the clinical impact of the tDCS intervention in a population including the DRE people of the present study, quantified the presence and duration of interictal epileptiform activity in the 1-h EEG recordings. Since no relevant changes were induced by the tDCS intervention (Assenza et al., 2017a), we decided not to devote a specific analysis to manage such periods in the connectivity analysis.

For the two stimulation sessions (Sham and Real, 1 month apart), we counted the seizure frequency in the week immediately preceding and following the stimulation. Stimulation-related seizure frequency changes were compared using the Wilcoxon sign rank test. We conducted a similar analysis to compare changes of interictal epileptiform activity in the present subgroup of persons. Spearman’s test was used to investigate the correlation between seizure frequency change and numbers of changed connections in each patient.

## Results

In each patient, Real ctDCS changed the brain FC (**Figure 1B**). After Sham, no FC change emerged in any patient. In all patients, FC changes induced by ctDCS involved the EF region, which was one node in the 73 ± 24% of changed connections across patients (**Table 2**). Within every single subject, there was a high consistency of the direction of FC changes (increase or decrease; red or blue connectors in **Figure 1B**, respectively) across all the analyzed EEG frequency bands (92 ± 12%). FC increased overall in five out of six patients, while in one it reduced. In the present group of patients, seizure frequency was clearly reduced by ctDCS (74.1 ± 41.2%) respect to Sham (39.4 ± 45.6) although it did not reach the threshold for statistical significance (*p* = 0.068). This was probably due to the small sample size. In fact, this sample of patients represents a subgroup of a slightly larger group of patients recently described (Assenza et al., 2017a), in which the ctDCS effect on seizure frequency was significant.

**Table 2:** Clinical and functional connectivity changes after ctDCS.

PT	Seizures			Functional connectivity changes					
	Pre-ctDCS	Post-ctDCS	Pre–post	TOTAL	Epileptic focus	Epileptic focus (%)	Epileptic focus (theta)	Epileptic focus (alpha)	Epileptic focus (beta)
DRE1	1	1	0	3	3	100	0	2	1
DRE2	1	0	1	-51	-28	55	-1	-19	-8
DRE3	1	0	1	4	3	75	0	3	0
DRE4	10	0	10	29	12	41	3	0	9
DRE5	100	3	97	15	16	100	8	7	1
DRE6	27	13	14	128	88	69	12	25	51
Median	5.5	0.5	5.5	9.5	14.0	73	1.5	2.5	1
Range	[1, 100]	[0, 13]	[0, 97]	[-51, 128]	[3, 88]	24	[-1, 12]	[-19, 25]	[-8, 51]


*Number of the seizures and of the connections before and after ctDCS. Functional connectivity changes are shown in the whole band and in the specific frequency bands (theta, alpha, and beta). PT, patient; TOTAL, among all regions; epileptic focus, one of the two nodes of the changed connection is the epileptic macro-region; epileptic focus (%), percentage with respect to the TOTAL changed connections; DRE, drug-resistant epileptic patient.*

**FIGURE 2:**
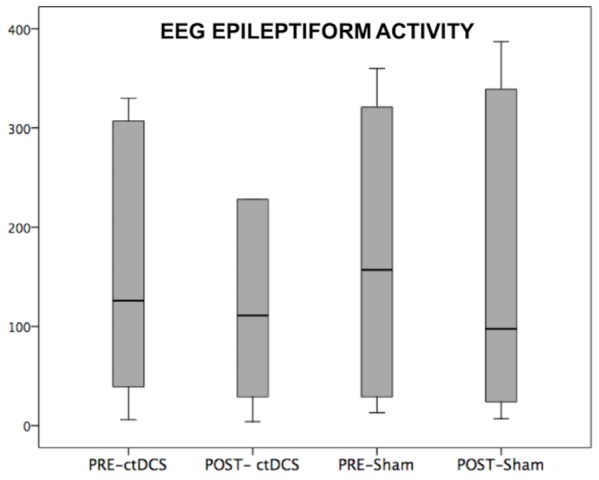
CtDSC-induced changes of EEG epileptiform activity. EEG epileptiform activity recorded in a 1-h EEG pre- and post-stimulations.

Despite the small sample size, a clear correlation appeared between the number of seizure changes and the changed connections of the EF region across all frequency bands (Spearman’s ρ = 0.809, *p* = 0.051). Considering the FC change separately in single bands, the correlation appeared in the theta band (Spearman’s ρ = 0.809, *p* = 0.051). Furthermore, comparing by independent-samples Wilcoxon test the two groups with higher (DRE4,5,6) and lower (DRE1,2,3) clinical symptoms and amelioration, the FC differed in the whole and theta band (*p* = 0.046). Interictal epileptiform activity changes induced by stimulating sessions did not differ between ctDCS and Sham (*p* > 0.200, **Figure 2**).

## Discussion

We demonstrated that Real ctDCS produced a FC change within the brain network of tDRE patients, absent after Sham stimulation. Interestingly, the changes of FC involving the EF correlated with the reduction of the number of seizures.

Present results confirmed the efficacy of ctDCS to modify human FC as assessed by means of EEG activity. Previous studies demonstrated that tDCS at rest increases FC among EEG sensors close to the cathode in healthy humans (Polanía et al., 2011). fMRI studies confirmed that tDCS affects functional brain reorganization and its ability to bias cortical networks (Antonenko et al., 2017; D’Mello et al., 2017). Animal studies corroborated this idea illustrating that tDCS interferes with brain networks by coordinating the competitive interplay of local processes over the whole brain (Krause et al., 2017). Given that several neurological and psychiatric diseases exhibit specific dysfunctions of brain’s FC (Zhang and Raichle, 2010), a tailored stimulation of the affected cortex can play an important role as a complement of standard therapy. Stroke, depression, and schizophrenia are exemplar brain disorders in which FC dysfunctions were reliably characterized and, actually, are the major object of neurostimulation clinical studies (Hasan et al., 2016; Assenza et al., 2017c; Loo et al., 2017).

The novelty of our results relies in the fact that we firstly report that ctDCS is able to modify FC in epileptic patients. This is extremely relevant in the light of the recent acquisition that epilepsy can be considered as a network disorder and not merely a focal disorder. In particular, fMRI, EEG, magnetoencephalography, and electrocorticography studies in focal epilepsies have demonstrated patterns of increased connectivity strictly around the epileptogenic zone. On the other hand, the connectivity of the epileptogenic zone with widespread distal networks seems reliably reduced in focal epileptic patients and correlated with neurocognitive problems. This FC decrease is also related to the duration and severity of epilepsy and is more evident in DRE patients respect to drug-responsive patients (Kay et al., 2013). However, it is not clear if FC decrease is caused by the destructive consequences of recurrent seizures, or an adaptive mechanism to prevent epileptic activity diffusion. Nevertheless, this FC reduction is a dynamic and reversible condition correlated with epileptic manifestations. Actually, the introduction of an efficacious pharmacological treatment is able to normalize FC alterations together with the disappearance of seizures (Clemens et al., 2014). Furthermore, in post-surgical tDRE patients, enhanced FC involving the area of EF resection occurs in parallel with a good seizure outcome (Englot et al., 2015). Our data seem to go in the same direction of this literature. In fact, the FC changes we recorded in our patients were mainly in the increase direction, in a population with a strong reduction of seizure frequency. In particular, the three patients with the highest number of seizure reduction displayed the major increase of FC. Furthermore, despite the small number of patients, a relevant correlation appeared between seizure reduction and FC changes of the EF. A network synchronization increase could sound harmful in epileptic patients; in particular if it involves the network of the region containing the EF as in our experiment. The safety of our data is supported by the fact that ctDCS did not produce an increase of seizures in any patient. Moreover, the lack of a ctDCS effect on EEG interictal epileptic activity suggests that FC modifications do not increase the synchronization of epileptic network *per se*. Thus, our data conceivably indicate that the ctDCS-induced FC increase rather relates a restoring of physiological brain connectivity to the detriment of epileptic activity propagation. This hypothesis is strengthened by the evidence (in our study and in previous literature) that the FC increase greatly involved background rhythms (alpha and beta) (Assenza et al., 2017c).

The correlation of the clinical and brain organization changes appeared considering the whole band neuronal activity. When assessing the FC expressed between neuronal oscillations in specific frequency bands, the correlation appeared in the theta band. This result could find an explanation in the type of FC we assessed. In facts, we mainly analyzed long-range connections which are known to be supported by slow (theta) activity, in opposite to local synchronization mainly mediated by higher (gamma frequency) oscillations (Canolty et al., 2006). Thus, we can conceive that ctDCS positively impinged the communication of EF with the whole brain.

We think that present results document that transcranial current stimulation is a promising technique to counteract pathological network activity in epileptic patients. We are conscious that there is lots of work to do in this direction but new frontiers of transcranial electric stimulation (tES) are at the window and could boost this innovative research field. Actually, we recently documented that a transcranial electric stimulation tailored on the endogenous neurodynamics of the target region [transcranial Individual neuroDynamics Stimulation (tIDS) (Cottone et al., 2018)] is superior respect to a standard tES technique in inducing a cortical neuromodulation in individual persons. Briefly, tIDS application needs a preliminary EEG-based network identification tool, called functional source separation (FSS) (Tecchio et al., 2007), which extracts the network’s electric potential associated with the selected activity (e.g., epileptic activity) and offers its dynamic features to build a customized neurostimulation to inhibit that specific network.

This scenario opens a new era in the treatment of epilepsy, which has in the electrical dysfunction the core of its pathophysiology, and in all neural pathological networks.

A main limitation of our study relates to our sample size. While we executed a reliable statistical analysis on the single-subject level assessing the FC changes with a solid base via the 1-h recording before and after Real and Sham stimulations, the small sample size is a big limitation in assessing the relationship between clinical and brain organization changes. Furthermore, the lack of a healthy control group does not allow confirming whether the direction of recorded changes goes toward a more physiological connectivity. A healthy control group could also test the specificity of the reported FC changes in epileptic patients. The acquisition of data in wider populations can open the use of EEG-based FC estimate as marker of therapeutic intervention efficacy.

## Conclusion

We reported innovative results about FC changes induced by ctDCS in tDRE patients, where the increased long-range FC of the EF correlated with the reduction of seizures.

## Author Contributions

GA contributed to conception and design of the study. GA, CC, CP, and AC acquired and analyzed the data. GA, FT, and VDL drafted and finally revised the manuscript and the figures.

## Conflict of Interest Statement

The authors declare that the research was conducted in the absence of any commercial or financial relationships that could be construed as a potential conflict of interest.

## Footnotes

1 http://www.uzh.ch/keyinst/NewLORETA/LORETA01.htm
